# Study protocol of a randomised clinical trial testing whether metacognitive training can improve insight and clinical outcomes in schizophrenia

**DOI:** 10.1186/s12888-020-2431-x

**Published:** 2020-01-29

**Authors:** Javier-David Lopez-Morinigo, Verónica González Ruiz-Ruano, Adela Sánchez Escribano Martínez, María Luisa Barrigón Estévez, Laura Mata-Iturralde, Laura Muñoz-Lorenzo, Sergio Sánchez-Alonso, Antonio Artés-Rodríguez, Anthony S. David, Enrique Baca-García

**Affiliations:** 1 Departamento de Psiquiatría, IIS-Fundación Jiménez Díaz, Madrid, Spain; 2 Departamento de Psiquiatría, Universidad Autónoma de Madrid, Madrid, Spain; 3 Centro de Especialidades Pontones, Salud Mental, 2ªPlanta, Ronda de Segovia, 52, 28005 Madrid, Spain; 4 Departamento de Teoría de Señal y de la Comunicación, Universidad Carlos III, Madrid, Spain; 5 Institute of Mental Health, University College London, London, UK; 6 Department of Psychiatry, University Hospital Rey Juan Carlos, Mostoles, Spain; 7 Department of Psychiatry, General Hospital of Villalba, Madrid, Spain; 8 Department of Psychiatry, University Hospital Infanta Elena, Valdemoro, Spain; 9 CIBERSAM (Centro de Investigación en Salud Mental), Carlos III Institute of Health, Madrid, Spain; 10 Universidad Católica del Maule, Talca, Chile

**Keywords:** Schizophrenia spectrum disorders, Metacognitive training, Insight, Ecological momentary assessment

## Abstract

**Background:**

Although insight in schizophrenia spectrum disorders (SSD) has been associated with positive outcomes, the effect size of previous treatments on insight has been relatively small to date. The metacognitive basis of insight suggests that metacognitive training (MCT) may improve insight and clinical outcomes in SSD, although this remains to be established.

**Methods:**

This single-center, assessor-blind, parallel-group, randomised clinical trial (RCT) aims to investigate the efficacy of MCT for improving insight (primary outcome), including clinical and cognitive insight, which will be measured by the Schedule for Assessment of Insight (Expanded version) (SAI-E) and the Beck Cognitive Scale (BCIS), respectively, in (at least) *n* = 126 outpatients with SSD at three points in time: i) at baseline (T0); ii) after treatment (T1) and iii) at 1-year follow-up (T2). SSD patients receiving MCT and controls attending a non-intervention support group will be compared on insight level changes and several clinical and cognitive secondary outcomes at T1 and T2, whilst adjusting for baseline data. Ecological momentary assessment (EMA) will be piloted to assess functioning in a subsample of participants.

**Discussion:**

To the best of our knowledge, this will be the first RCT testing the effect of group MCT on multiple insight dimensions (as primary outcome) in a sample of unselected patients with SSD, including several secondary outcomes of clinical relevance, namely symptom severity, functioning, which will also be evaluated with EMA, hospitalizations and suicidal behaviour.

**Trial registration:**

ClinicalTrials.gov: NCT04104347. Date of registration: 26/09/2019 (Retrospectively registered).

## Background

Schizophrenia and related disorders remain associated with relatively poor psychosocial outcomes [1]. Impaired insight has been reported to be the strongest predictor of this poor outcome in psychotic disorders [2]. However, the effect size of previous treatments on insight changes in psychotic disorders has been relatively small to date [3, 4], which may have been the result of not tackling the actual underpinnings of insight in psychosis.

Several theories have been proposed to explain what underlies ‘lack of insight’ in schizophrenia spectrum disorders. First, lack of insight could be viewed as having a function in terms of being protective or preserving self-esteem, i.e., a denial mechanism [5]. Second, lack of insight may also be considered as a primary symptom of the disorder [6]. Third, the neurocognitive basis of insight was in part supported by a meta-analysis [7], which also suggested that other variables, namely metacognition, which was defined as ‘the ability to think of one’s and others’ thinking’ [8, 9], may affect insight. Indeed, patients with schizophrenia have been reported to show metacognitive deficits [10, 11] and poorer metacognitive performance is linked with impaired insight in schizophrenia [12]. Metacognitive training (MCT) may therefore improve insight, which should also have a positive impact on clinical outcomes.

MCT was first developed by Steffen Moritz and Todd Woodward [13] and is available at no cost at: http://www.uke.de/mct. Since then, one systematic review [14] and four meta-analyses [15–18] have replicated the positive effects of MCT on positive symptoms, particularly delusions, when compared with ‘treatment as usual’ (TAU), although one meta-analysis [19] failed to show such an association. However, to the best of our knowledge no definitive randomised clinical trial (RCT) using MCT has considered insight as primary outcome to date, although three previous RCTs found other non-MCT metacognitively oriented therapies to improve insight in first-episode psychosis [20] and schizophrenia [21, 22].

Clinical and cognitive insight are different, albeit related, concepts [2, 12]. There has been a growing interest in clinical insight in psychosis since the multidimensional model of insight proposed by David [23], which encompasses three different, albeit overlapping, dimensions - illness recognition, symptom relabelling and treatment compliance – and has been consistently replicated ever since [24]. Multidimensional measurement scales, such as the Scale of Unawareness of Mental Disorder (SUMD) [25] and the Schedule for Assessment of Insight (SAI-E) [26], were also devised for research. Cognitive insight is a core metacognitive domain which refers to the person’s ability to evaluate and correct his/her own distorted beliefs and misinterpretations (self-reflectiveness) and the tendency to overconfidence in one’s conclusions (self-certainty) [10].

The ***main aim*** of this RCT is to test whether MCT can improve insight, including clinical and cognitive insight, in patients with schizophrenia over a 1-year follow-up. As ***secondary aims,*** we will investigate the effect of MCT-related insight changes on clinical outcomes, including symptomatic severity, hospitalizations, suicidal behaviour and psychosocial functioning. In addition, we will pilot the use of ecological momentary assessment (EMA) via two web-based applications - www.MEmind.net [27] and the Evidence-Based Behaviour platform eB2 app [28] - to measure functioning in a subsample of participants.

Specifically, the following five hypotheses are to be tested: i) that MCT will result in higher cognitive and clinical insight levels and that MCT-induced insight improvement will be linked with ii) reduced symptom severity and iii) hospitalizations, iv) lower suicide rates and v) better functioning than in controls.

## Methods

### Study design

This is a single-center, assessor-blind, parallel group, two-armed randomised controlled trial (RCT) over a 1-year follow-up period. Participants will be assessed at baseline (T0), following which they will be randomised to either group MCT (experimental group) or a support group (control group) and they will be reassessed after treatment, i.e., at approximately 8 weeks (T1), and at 1-year follow-up (T2).

The study protocol has been approved by the local Research Ethics Committee of the Instituto de Investigación Sanitaria (IIS)-Fundación Jiménez Díaz (Madrid, Spain), from which patients will be recruited as detailed below, and registered as RCT-EC044-19_FJD_HRJC. Also, the protocol is registered at ClinicalTrials.gov (NCT04104347).

### Sample and eligibility criteria

The sample comes from the Centro de Salud Mental de Arganzuela, which is an outpatient clinic linked with Hospital Universitario Fundación Jiménez Díaz (FJD), which provides publicly-funded medical and mental healthcare to approximately 500,000 inhabitants residing in our geographic catchment area in Madrid (Spain). Those adults (age 18–64 years) with a confirmed diagnosis of psychosis according to the Mini International Neuropsychiatric Interview, 5th Edition (MINI) [29], will be invited to participate in the study. The exclusion criteria will be as follows:

- IQ ≤ 70 as measured by the short form of the Wechsler Adults Intelligence Scale (WAIS)-IV [30].
- History of head injury and/or a neurological condition.
- Having received a metacognitively oriented therapy within the previous year.
- Low level of Spanish.
- Lack of cooperativeness with the assessment and/or “intervention”.

Therefore, the initial recruitment is unselected, that is, all adults (age 18–64 years, both inclusive) with a confirmed diagnosis of schizophrenia spectrum disorder receiving mental healthcare in our outpatient clinic (hence, clinically stable – i.e., not acutely unwell -) could be included in the study, provided they do not meet any of the above exclusion criteria. This approach has, of course, advantages and disadvantages, which are discussed further in the section of discussion. Those who agree to take part in the study will be asked to give written informed consent.

### Recruitment process

Recruitment will occur at the outpatient clinic known as Centro de Salud Mental de Arganzuela at which two consultant psychiatrists (LMI, SSA) and one consultant psychologist (LML) will refer potential candidates to the principal investigator of the project (JDLM) to arrange a first pre-recruitment interview during which the relevant information of the project is explained to the candidate in lay terms, including an information leaflet. Those agreeing to participate in the study are screened against the above inclusion/exclusion criteria at this point. Those who are found to fulfill the study selection criteria undertake a neurocognitive assessment. Specifically, the WAIS-IV [30] is administered by a MSc-level clinical psychologist (VGRR) in order to rule out an IQ ≤ 70, which is an exclusion criterion. Those with an IQ > 70 are administered the Trail Making Test (TMT) [31], which assesses executive function and has been found to correlate with insight scores in psychosis patients [32], by VGRR shortly after the WAIS-IV in order to complete the baseline neurocognitive assessment at this point.

After a short break of around 5 min, JDLM performs the MINI [29] to confirm the diagnosis of psychosis and conducts the psychopathological, insight, metacognitive and functioning assessments detailed below. Also, a set of demographic and clinical variables, which are listed below, are collected at this baseline interview.

All participants will be encouraged to continue taking medication as prescribed by the treating consultant psychiatrist, usually antipsychotics, and to receive non-metacognitive oriented psychotherapy, such as psychoeducation. There will be no restrictions on medication changes over the trial, which can be made by the treating consultant at any time, although medication-related variables will be considered in secondary analyses as potential mediators/confounders. Participants are informed of their right to drop out of the study at any time without having to disclose a specific reason, which will have no implications on treatment or service provision. Participants are not compensated with any amount of money for completing the assessments and/or receiving the interventions, namely MCT or attending a weekly support group.

### Randomisation and assessor-blindness

**Fig. 1:**
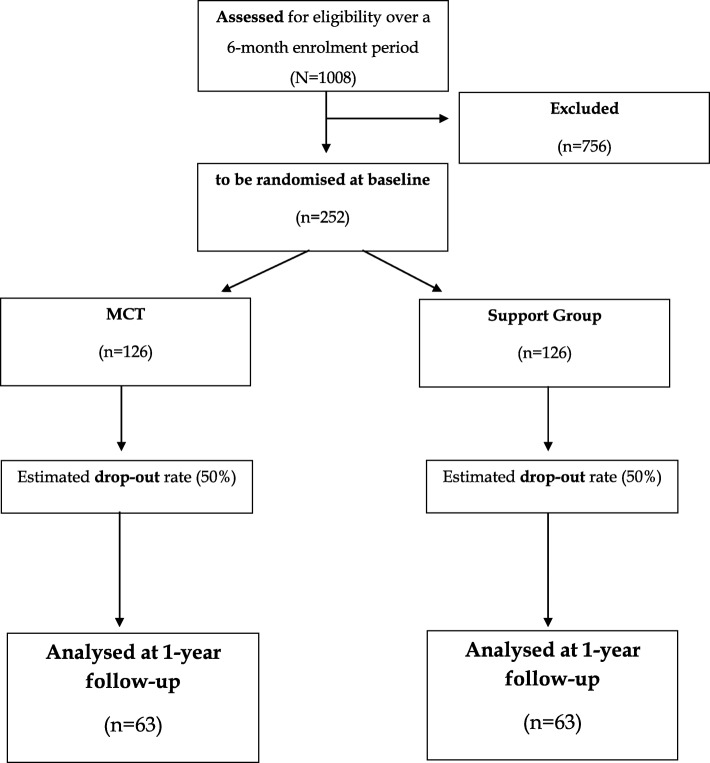
Study CONSORT chart. Estimated flow of participants over the trial period

After the baseline (T0) assessment participants are randomised to either MCT or the support group, both of which are run by VGRR and ASEM, through a computerized plan (no stratification factors) in blocks of 10 subjects (maximum number of each group) and assessor (JDLM)-patient blind. Only one co-principal investigator of the project (EBG) has access to the randomisation plan, but the assessor (JDLM) is not informed of the patient’s allocation group, thus ensuring assessor-blindness since the patient may find out what intervention he/she is receiving, for which the study cannot be considered to be double-blind, although it is ‘single-blind’, that is, ‘assessor-blind’. Also, big efforts will be made on the ground to reduce the risk that the assessor (JDLM) may become accidentally unblinded and patients are reminded that they should not disclose their group assignment at any time. At the end of the baseline assessment, participants receive an envelope with the intervention assignment from an independent administrator who is not involved in the research team. A reminder mobile text-message is also sent to participants within the next 24 h with the group details (date, time and venue), which is also re-sent again during the 24 h prior to the appointment, which is very similar to our routine clinical practice. Hence, assessor-blindness is guaranteed at all times throughout the study period since the assessor (JDLM) is not involved in the randomization plan or the active interventions. See the CONSORT flowchart in Fig. 1, below, for details.

### Assessments

Participants will be assessed at three timepoints: i) T0: at baseline; ii) T1: after treatment; iii) T2: at 1-year follow-up. Data on different variables will be collected at each of these assessments, which is detailed in the SPIRIT flowchart shown in Table 1, below.

**Table 1:**
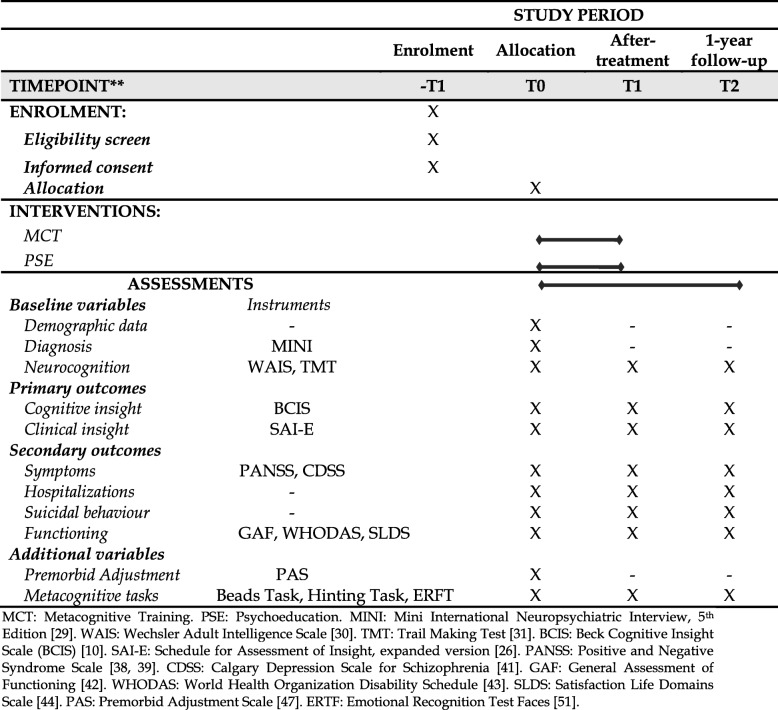
SPIRIT flowchart

*MCT* Metacognitive Training*. PSE* Psychoeducation. *MINI* Mini International Neuropsychiatric Interview, 5th Edition [29]. *WAIS* Wechsler Adult Intelligence Scale [30]. *TMT* Trail Making Test [31]. *BCIS* Beck Cognitive Insight Scale (BCIS) [10]. *SAI-E* Schedule for Assessment of Insight, expanded version [26]. *PANSS* Positive and Negative Syndrome Scale [38, 39]. *CDSS* Calgary Depression Scale for Schizophrenia [41]. *GAF* General Assessment of Functioning [42]. *WHODAS* World Health Organization Disability Schedule [43]. *SLDS* Satisfaction Life Domains Scale [44]. *PAS* Premorbid Adjustment Scale [47]. *ERTF* Emotional Recognition Test Faces [51]

Both raters (JDLM and VGRR) completed a rater-training workshop and received individual training in which they were observed as they assessed pilot patients by using the project scales explained below under the supervision of a senior consultant psychiatrist with expertise in the use of these instruments (EBG).

### Outcome measures

#### Co-primary outcomes

Insight is the primary outcome of this RCT and both clinical and cognitive insight will be considered.

- *Clinical insight* will be measured with the Spanish version [33] of the Schedule for Assessment of Insight, expanded version (SAI-E) [26]. The SAI-E takes the form of a semi-structured interview which enquires about different aspects of insight and provides scores on three insight dimensions in line with David’s model [23] - illness recognition, symptom relabeling, treatment compliance - and a total insight score. The scale was found to be easily applicable in routine clinical practice [34] and good to excellent inter-rater reliability was reached, with total insight scores intra-class correlations coefficients ranging from 0.92 to 0.98 (*p* < 0.001) [35]. JDLM received training from the scale author (ASD) and they both co-led the validation study of the SAI-E Spanish version [33], which will be used in this RCT.
- *Cognitive insight* will be assessed by the Spanish version [36] of the Beck Cognitive Insight Scale (BCIS) [10], which is a 15-item self-administered scale which evaluates self-reflectiveness (9 items) and self-certainty (6 items). A composite index can thus be calculated by subtracting self-certainty from self-reflectiveness. Internal consistency was found to be acceptable (Cronbach’s α = 0.60–0.68) [37].

#### Secondary outcomes

Secondary outcomes of this investigation include symptomatic severity, hospitalizations, suicidal behaviour, metacognition, neurocognition and functioning.

##### Symptomatic severity

Symptoms severity will be rated with the Spanish version [38] of the Positive and Negative Syndrome Scale (PANSS) [39]. Based on a systematic review of previous PANSS factor analyses [40], five symptomatic dimensions will be analysed: positive, negative, disorganization, mania and depression, which will also be measured with the Calgary Depression Scale for Schizophrenia (CDSS) [41].

##### Hospitalizations

Number of hospitalizations*,* i.e., time to hospitalization (i.e., survival analyses, see statistical analyses below), and total number of days in hospital will be considered. Mental health presentations to the emergency department will be included in secondary analyses.

##### Suicidal behaviour

In order to investigate the effects of MCT on suicidal behaviour, time to first suicidal event, including suicide attempts and suicide completions, over the 1-year follow-up study period will be investigated.

##### Functioning

Functioning will be recorded through the Global Assessment of Functioning (GAF) [42], the World Health Organization Disability Schedule (WHODAS) [43] and the Satisfaction Life Domains Scale (SLDS) [44]. In addition, as a relatively novel methodology in patients with psychotic disorders, ecological momentary assessment (EMA) [27] will be piloted in a subsample of those participants who agree to set up two web-based applications in their smartphones, namely Memind (www.memind.net) and eB2 [28]. Memind is an ‘active’ platform which has two different interfaces or views, namely staff and patient view. The ‘electronic health record’ allows staff to electronically record data on sociodemographic, diagnostic and pharmacological variables, while the patient view was designed in order for patient to self-rate some scales such as the BCIS [10], which will be one of the primary outcome measures in this RCT as detailed above. On the other hand, Evidence-Based Behavior (eB2) is a ‘passive’ platform designed for recording functioning-related data, such as mobility, i.e., location, distance, speed; physical activity i.e., number of steps, sleep data; social activity, i.e., phone use, active apps, social network data and emotions, i.e., prosodic analysis of speech signals and text message emotion analysis), without the subject’s collaboration (it is ‘passive’). Data are anonymised and sent to a secure server. Memind and eb2 will be combined to create a machine learning mental health system allowing a continuous feedback from digital phenotyping [45].

#### Additional variables

##### Demographic and clinical data

At baseline data on the following demographic and clinical variables will be collected: gender, age at the study inception, nationality, ethnicity, marital status, education level, living status, employment status, ICD-10 diagnosis, duration of untreated psychosis (which will be estimated with the Nottingham Onset Schedule, [46], number of previous admissions, number, date and method of previous suicidal acts, current medications, alcohol/illicit drugs dependence (present/absent, medical comorbidities (present/absent) family history of mental illness (present/absent).

Marital status, living status and employment status will be reassessed at 1-year follow-up as measures of functioning.

##### Premorbid adjustment

Premorbid adjustment, which can be defined as ‘the degree of achievement of developmental goals’ will be retrospectively rated with the Premorbid Adjustment Scale (PAS) [47]. Specifically, the PAS provides scores on the level of adjustment over i) childhood (to age 11), ii) early adolescence (age 11–15), iii) late adolescence (age 15–17) and iv) adulthood (age ≥ 18). With regard to childhood and adolescence, items inquiry about sociability and social withdrawal, peer relationships, scholastic performance, adaptation to school and ability to form socio-sexual relationships. The questions regarding adulthood focus on social relationships by asking about educational achievement, social relationships and level of interest in, and enjoyment of, major life activities, such as work or family.

##### Neurocognition

The neurocognitive assessment battery, which will be administered at the three assessments, will include the short version of the Wechsler Adult Intelligence Scale (WAIS) Revised [30], which estimates current intelligence quotient (IQ), and the Trail Making Test (TMT) [31], which assesses executive function. Subtracting time in seconds to complete TMT task B minus time in seconds to complete TMT task A gives a measure of executive function, whilst controlling for processing speed.

##### Metacognition

Three metacognitive tasks will be completed by participants at the three assessments (T0, T1, T2):

- Jumping to Conclusions (JTC) will be determined with the beads task [48].
  During this task, the individual is asked to decide the jar to which the extracted bead belongs on the basis of probability (in task 1 the probability is 85:15, while in task 2 the probability is 60:40). ‘JTC’ is considered as making a decision after extracting one or two beads.
- The Hinting Task [49, 50] will be used to measure Theory of Mind (ToM) performance. Learning is avoided by using different stories at the three assessments. Cronbach’s α was good (0.64) for the Spanish version [37].
- In addition, the Emotional Recognition Test Faces [51], which is composed of 20 different pictures representing people’s emotions, will be used to evaluate ToM.

### Intervention

Participants will be randomised either to receive (in addition to their usual treatment, including antipsychotic medication) MCT or to attend a support group. Both ‘interventions’ are implemented for the same duration (one session per week lasting for about 45–60 min over 8 weeks). Unlike comparing MCT with ‘treatment as usual’ (TAU), controls will attend a weekly support group to avoid group attendance-related biases, consistent with some previous RCTs on MCT [37].

#### Metacognitive training (MCT)

Metacognitive Training (MCT) was first developed in Germany in 2007 by Steffen Moritz and Todd Woodward [13] targeting positive psychotic symptoms of patients with schizophrenia. In short, MCT seeks ‘to plant the seeds of doubt’ regarding cognitive biases leading to delusional thoughts rather than asking patients to talk about of their problems, which is likely to be distressful, particularly within the context of a group. MCT consists of ten group sessions focused on different topics (Modules) as follows: Attributional Style (Module 1), Jumping to Conclusions (Modules 2 and 7), Changing Beliefs (Module 3), Empathy (Modules 4 and 6), Memory (Modules 5), Depression and Self-Esteem (Module 8) and two additional modules, namely Self-Esteem (Module 9) and Stigma (Module 10). Modules 8 (Self-Esteem), 9 (Self-Esteem) and 10 (Stigma) can be delivered together as one session so the intervention totals eight weekly sessions, that is, 8 weeks. The therapists who will deliver MCT to participants of this RCT (VGRR and ASEM) undertook training from one of the co-authors of the Spanish version of the MCT manual (MLBE), which is available at: http://www.uke.de/mkt and was directly supervised by Steffen Moritz.

#### Support group

Controls will attend eight weekly support groups. In line with the ‘treatment as usual’ delivered to most patients with psychosis in our clinic, seven sessions will focus on the following topics: 1) basic activities of daily living (BADL), 2) instrumental activities of daily living (IADL), 3) physical health, 4) press-based work, 5) psychoeducation on emotions, 6) psychoeducation on illness, 7) social and family relationships. One additional session will give participants some time to openly raise general issues and concerns which were not discussed during the above sessions. In order to minimise the effect of this ‘intervention-like’ on controls, the interaction/input from the therapists (VGRR and ASEM) will be minimal. Thus, controls will be encouraged to attend these group sessions, from which they can benefit. While not an intervention as such, this should turn into enough incentive to reach similar attendance rates in both arms of the RCT.

Treatment fidelity will be looked at by recording a random selection of MCT sessions (after obtaining consent from participants), which will be independently assessed against the manual criteria (http://www.uke.de/mkt) by two researchers from our group (MLBE and EBG) with no involvement in the assessments or interventions. These two researchers will also determine whether controls may have been significantly exposed to elements of metacognition by accident during the group sessions.

In addition to the randomisation group intervention, all participants will have full access to their treatment so they are expected to continue taking medication and receiving non-metacognitive-based psychotherapies as recommended by the treating multidisciplinary team. This will also allow early identification of potential adverse events, unintended effects of the trial and trial misconduct.

### Statistical analysis

Participants’ data will be analysed at the end of the study on an intention-to-treat (ITT) basis, thus including all patients with baseline information available. Imputation methods will be used to estimate missing values. All the analyses will be performed with the Statistical Package for Social Science version 25.0 (SPSS Inc., Chicago, IL, USA).

First, demographic and clinical characteristics of groups (MCT and controls), including insight measures, will be compared at baseline so parametric and non-parametric tests will be used as appropriate. Also, for descriptive purposes, between-arm completion rates differences will be analysed at the end of the study.

Second, in order to investigate the primary outcome of the study, namely insight changes over the trial period, univariate analysis of covariance (ANCOVA) models will examine between-group differences (MCT and controls) in the SAI-E and BCIS total and subtotal scores changes from T0 to T1 and from T0 to T2 (as the dependent variable), whilst adjusting for baseline data. Hence, the effects of treatment (independent of time) and treatment group allocation*time interactions on insight changes will be investigated. We will also explore within-group scores changes between T0, T1 and T2. Specifically, we will estimate effect sizes (Cohen’s *d*) and the corresponding 95% confidence intervals (CI) from the imputed datasets for between- and within-group insight scores changes. In accordance with Cohen’s conventions [52], effect sizes will be classified as ‘small’ (*d < 0.2),* ‘medium’ *(d* = 0.2–0.5) or ‘large’ (*d* > 0.8). Due to multiple testing and the subsequent risk of type I error, results will be adjusted using Bonferroni correction.

Third, for the secondary outcomes evaluated with continuous variables, i.e., symptom severity (PANSS and CDSS) and functioning (GAF, SDLS and WHODAS), analogous ANCOVA models will be used. For those binary secondary outcomes, namely suicidal behaviour and readmissions, survival analyses, i.e., multivariable Cox regression models [53], will model time to the outcome event (i.e., first suicidal event or hospital admission, respectively) or the censoring date as appropriate, whilst adjusting for baseline variables.

Finally, variables inter-relationships over the trial period will be examined by means of path analysis through structural equation modeling, thus testing the effect of putative mediators/moderators/confounders/covariates, including neurocognition measures, on the above associations.

#### Power calculations and estimation of sample size

Given that the mean SAI-E score for psychosis patients is 13/28 with a standard deviation of around 6 [32, 35], a difference of 2 points (e.g., 13 vs. 15) between groups (e.g., MCT vs. controls), which is considered to be clinically meaningful [26], is equivalent to an effect size of 0.33 with a two-tailed alpha significant level set at 5%. Under these assumptions, for reaching a sufficient statistical power of β = 80% at the end of the study, we will need *n* = 63 subjects in each arm, that is, a total sample size of *N* = 126 patients, who will be analysed at the end of the study on an intention-to-treat (ITT) basis. Attrition rates in previous RCTs investigating MCT effects on symptoms were low (approximately 10%) [18]. However, since we will follow-up patients over a more prolonged period (1 year) we have conservatively assumed a much higher drop-out rate of 50%. Under this assumption, the study will be carried out with an initial sample size of *N* = 252 patients, i.e., *n* = 126 participants in each group/arm at the study inception, which will also allow both ITT and ‘per protocol’ analyses with sufficient power. See the CONSORT flow chart in Fig. 1 for details.

## Discussion

### Relevance and impact

This is the first large RCT testing the long-term effects of group MCT on clinical and cognitive insight in addition to several relevant clinical outcomes in a representative sample of patients with schizophrenia spectrum disorders. Although MCT has been consistently demonstrated to improve positive symptoms [14–18], the benefits of MCT on insight are yet to be established [3], which is of major clinical relevance given the association of insight with outcomes in schizophrenia and related disorders [2]. This is supported further by three previous trials which revealed other non-MCT metacognitively oriented treatments to have a positive impact on insight [20–22].

The clinical relevance will be immediate in raising clinicians’ awareness of insight, particularly regarding its role in clinical outcomes. In addition, this RCT will add to the field by showing the possibility of improving insight through an eassily applicable intervention such as MCT, which represents a more patient-centred treatment approach.

### Outcome measures

In addition to the primary outcome of this RCT, namely insight, we will consider several clinical outcomes, both positive and negative, such as symptom severity, functioning, readmissions and suicidal behaviour, thus testing the so-called ‘Insight Paradox’ [54]. Although the positive impact of MCT on symptoms has been established [14–18], most of previous RCTs included in these meta-analyses compared MCT with TAU. Therefore, not only this RCT may replicate this, but also by comparing the MCT group with an active intervention control group, if the above positive effect was shown, this would provide further support for the benefits of MCT on symptoms. Beyond and above the effects of MCT on symptoms, functioning will be comprehensively examined, consistently with current guidelines focused on recovery as main outcome measure [55]. Also, functioning will be evaluated with novel methodology such as EMA, which has not been sufficiently researched in psychosis patients.

However, insight was also linked with negative outcomes. In particular, insight was thought to lead to depression, hopelessness and suicidality, which is known as the demoralization syndrome [56, 57] or ‘Insight Paradox’ [54]. However, recent research did not show a ‘direct’ relationship between insight and suicidality in first-episode psychosis, which was due to the confounding effects of previous suicide attempts and depression on such an association [32]. The question therefore arises, i.e., whether an insight improving intervention (such as MCT, which is to be tested in this trial) may reduce suicide risk (and improve mood) in psychosis, which will be properly investigated in this RCT. In addition to suicidal behaviour-related outcomes, we will test whether MCT can reduce admissions over the trial period, which is of relevance from a cost-effectiveness perspective [58].

### Methodological issues

Insight, which is the primary outcome of the study, will be measured with the multidimensional SAI-E scale [26] based on the multidimensional David’ model of insight [23]. This will allow us to look at the effects of MCT on insight dimensions individually, which may differ.

Recruitment and retention of patients in psychosis research is challenging, particularly in long-term studies. Accordingly, given our 1-year follow-up study period we have decided to invite all those potentially eligible patients who are seen at our clinic during the 6-month enrolment period. This approach has, of course, strengths and weaknesses. On the one hand, the sample will be very likely to be representative of the local population of psychosis patients, most of whom are, to some degree, chronic (rather than early-onset psychosis patients). On the other hand, the inclusion of chronic patients may reduce the room for improvement. However, it is precisely this group of patients, which tends to be relatively neglected by research and stakeholders in comparison with first-episode patients, who may particularly benefit from receiving MCT. Moreover, this design will allow us to examine whether chronicity, including (mild) cognitive impairment, mediates the response to MCT, i.e., by comparing response to MCT (in comparison with controls) in chronic and early-onset patients. Nevertheless, those with an IQ ≤ 70 will be excluded from the study.

Unlike most of previous studies, both controls and the intervention group (MCT) will attend similar weekly group sessions at the clinic with support from the clinical team, thus minimising stigma-related issues. When designing the study with an active control group, we were aware that we were reducing the room for improvement, that is, the likelihood of finding between-group differences in terms of insight and the other secondary outcomes of interest, namely symptom severity, admissions, suicidal behaviour and functioning. However, this design will represent a more challenging test for MCT to demonstrate whether it should be incorporated into routine clinical practice and guidelines. Thus, this approach will make the trial more similar to our routine practice since patients regularly attend psychoeducation-like/supportive groups at our clinic at staff (psychiatrists and psychologists) discretion on the basis of regular individualised needs assessments, which may also maximise completion rates among controls. Nevertheless, participants will be reminded via mobile text message their next group appointment within the previous 24 h. In addition, it is worth noting that from an ethical perspective, although controls will be prevented from receiving MCT, at least they will be able to attend a weekly supportive group at the outpatient clinic, from which they may benefit in addition to their treatment as usual.

With regard to the statistical analyses, two points deserve some comment. First, the effect of variables changes over time on outcome measures will be considered since survival analyses will be performed. Second, by using path analysis we will be able to better understand the causality relationships between the variables tested in the models, which is what really matters. In addition, devising new dynamic probabilistic models to extract knowledge in the form of digital biomarkers obtained jointly from the patient’s EMA and electronic health records should contribute to paving the way towards the so-called personalized medicine.

### Strengths and weaknesses

At this pre-recruitment stage, we already acknowledge that this RCT has strengths and weaknesses. In particular, we intend to recruit a large sample size of SSD patients who will also be representative of our local population since most of our psychosis patients receive mental healthcare in our clinic with the exception of a tiny proportion in either primary care or in the private sector. As detailed above, controls will also attend a weekly ‘therapeutic-like’ group, thus controlling for the effect of attending a group, which will represent a more challenging test for MCT. In addition, the main authors of MCT, Steffen Moritz and Todd Woodward, will not get directly involved in this RCT, thus avoiding allegiance-related biases. Of course, the vast majority, if not all, patients are expected to continue taking medication as prescribed, particularly antipsychotics, so medication-related variables will be included in secondary analyses since they may affect the effect of MCT on insight.

However, this is a RCT so participants are required to give consent and complete a comprehensive set of assessments. Hence, those with the lower levels of cooperativeness, and therefore with poorer insight, are not likely to take part in this study, which may limit the generalisability of the results. Nevertheless, this ethical requirement, and the subsequent limitation in terms of generalisability, applies to most studies on insight in psychosis.

## Conclusions

To sum up, to the best of our knowledge this will be the first RCT investigating the effect of group MCT on multiple dimensions of cognitive and clinical insight (as co-primary outcomes) in a real-world sample of unselected patients with SSD. Also, the impact of these potential MCT-related insight changes on several secondary clinical outcomes, such as symptom severity, functioning, which will be also assessed with EMA, hospitalizations and suicidal behaviour, will be looked at.

## Data Availability

Data from this RCT will be available for further analyses on request provided rules on use of the RCT database (as detailed by the ethical approval) are complied with.

## Abbreviations

BCIS: Beck Cognitive Insight Scale
CDSS: Calgary Depression Scale for Schizophrenia
EMA: Ecological Momentary Assessment
ERTF: Emotional Recognition Test Faces
FJD: Hospital Universitario Fundación Jiménez Díaz
GAF: General Assessment of Functioning:
IQ: Intelligence Quotient
JTC: Jumping to Conclusions
MCT: Metacognitive training
MINI: Mini Internacional Interview
PANSS: Positive and Negative Syndrome for Schizophrenia
PAS: Premorbid Adjustment Scale
RCT: Randomised clinical trials
SAI-E: Schedule for the Assessment of Insight, expanded version
SLDS: Satisfaction Life Domains Scale
SSD: Schizophrenia spectrum disorders
TMT: Trail Making Test
WAIS: Wechsler Adult Intelligence Scale
WHODAS: World Health Organization Disability Schedule
